# A nomogram model based on SII, AFR, and NLR to predict infectious complications of laparoscopic hysterectomy for cervical cancer

**DOI:** 10.1186/s12957-024-03489-0

**Published:** 2024-07-24

**Authors:** Hailin Xing, Donglan Yuan, Yabin Zhu, Lin Jiang

**Affiliations:** 1 Department of Anesthesiology, The Affiliated Taizhou People’s Hospital of Nanjing Medical University, Taizhou School of Clinical Medicine, Nanjing Medical University, Taizhou City, China; 2 Department of gynecology,The Affiliated Taizhou People’s Hospital of Nanjing Medical University, Nanjing Medical University, Taizhou School of Clinical Medicine, Nanjing Medical University, No. 366 Taihu Road, Taizhou City, 225300 Jiangsu Province China

**Keywords:** Cervical cancer, Laparoscopic hysterectomy, Postoperative infectious complications, Risk factor, Nomogram model

## Abstract

**Background:**

This study aimed to investigate the potential risk factors associated with postoperative infectious complications following laparoscopic hysterectomy for cervical cancer and to develop a prediction model based on these factors.

**Methods:**

This study enrolled patients who underwent selective laparoscopic hysterectomy for cervical cancer between 2019 and 2024. A multivariate regression analysis was performed to identify independent risk factors associated with postoperative infectious complications. A nomogram prediction model was subsequently constructed and evaluated using R software.

**Results:**

Out of 301 patients were enrolled and 38 patients (12.6%) experienced infectious complications within one month postoperatively. Six variables were independent risk factors for postoperative infectious complications: age ≥ 60 (OR: 3.06, 95% confidence interval (CI): 1.06–8.79, *P* = 0.038), body mass index (BMI) ≥ 24.0 (OR: 3.70, 95%CI: 1.4–9.26, *P* = 0.005), diabetes (OR: 2.91, 95% CI: 1.10–7.73, *P* = 0.032), systemic immune-inflammation index (SII) ≥ 830 (OR: 6.95, 95% CI: 2.53–19.07, *P* < 0.001), albumin-to-fibrinogen ratio (AFR) < 9.25 (OR: 4.94, 95% CI: 2.02–12.07, *P* < 0.001), and neutrophil-to-lymphocyte ratio (NLR) ≥ 3.45 (OR: 7.53, 95% CI: 3.04–18.62, *P* < 0.001). Receiver operator characteristic (ROC) curve analysis indicated an area under the curve (AUC) of this nomogram model of 0.928, a sensitivity of 81.0%, and a specificity of 92.1%.

**Conclusions:**

The nomogram model, incorporating age, BMI, diabetes, SII, AFR, and NLR, demonstrated strong predictive capabilities for postoperative infectious complications following laparoscopic hysterectomy for cervical cancer.

## Introduction

Cervical cancer ranks as the fourth worldwide [1] with approximately 660,000 new cases and 350,000 deaths in 2022 by the World Health Organization (WHO). In China, cervical cancer is the fourth most common cancer among women and poses a significant threat to public health [2]. Laparoscopic radical hysterectomy is indeed a major treatment strategy for cervical cancer, particularly for early-stage disease. This minimally invasive surgical approach offers reduced morbidity, quicker recovery, and better cosmetic outcomes than traditional laparotomy [3].

However, postoperative complications have significant adverse impacts on the prognosis of patients after surgery for cervical cancer. Infection complications are very common postoperative complications after laparoscopic hysterectomy, associated with delayed recovery, extended hospital stays, increased costs, and even elevated postoperative morbidity [4]. Therefore, it is crucial to investigate the potential valid predictive factors for infectious complications to improve prognosis. Risk factors for the developing postoperative infectious complications include smoking, obesity, uncontrolled diabetes, compromised immune function, and prolonged hospital stays [5]. Despite ongoing efforts by the researchers to explore predictive factors, significant gaps remain in this area. This study aimed to investigate the potential predictors and construct a nomogram prediction model for postoperative infectious complications.

## Materials and methods

This retrospective cohort study included individuals who underwent consecutive selective laparoscopic hysterectomy for cervical cancer at The Affiliated Taizhou People’s Hospital of Nanjing Medical University, between 2019 and 2024. The inclusion criteria were as follows: (1) patients diagnosed with cervical cancer supported by pathological evidence; (2) patients who underwent laparoscopic hysterectomy under general anesthesia; and (3) patients with follow-up for at least one month. The exclusion criteria were as follows: (1) Incomplete clinical data or follow-up; (2) patients who underwent laparotomy or laparoscopic conversion to laparotomy; (3) patients who were recently treated with immunosuppressants; (4) patients with pre-existing infection before the surgery; (5) patients who underwent neoadjuvant therapy for advanced cervical cancer; (6) patients who underwent radiotherapy after the surgery. The study received approval from the hospital’s ethics committee, and all enrolled patients provided written informed consent.

The enrolled patients underwent surgery under general anesthesia for surgery, with induction achieved using sufentanil, propofol, rocuronium bromide, and midazolam. Anesthesia was maintained using sevoflurane, remifentanil, and dexmedetomidine. To prevent infection, the patients in the study were routinely administered antibiotics both pre- and post-surgery antibiotics. Furthermore, a drainage tube was consistently inserted before the completion of the surgical procedure.

The following data were collected: (1) Demographic information, including age, body mass index (BMI), and presence of pausimenia; (2) clinically relevant details, including pathological type, International Federation of Gynecology and Obstetrics (FIGO) stage, American Society of Anesthesiologists (ASA) score, comorbidities of hypertension, diabetes, chronic obstructive pulmonary disease (COPD), history of abdominal surgery, operation time, and lymph node dissection; (3) preoperative laboratory variables, including white blood cells (WBC), hemoglobin, platelets, C-reactive protein (CRP), albumin, fibrinogen, monocytes, neutrophils, and lymphocytes.

The primary outcome of this study was the incidence of infectious complications within one month post-surgery. The diagnosis of postoperative infectious complications (mainly including surgical site, respiratory, and urinary tract infections) typically follows clinical criteria that include a combination of symptoms (fever, tachycardia, and others), laboratory findings (white blood cells and others), sometimes urine and blood cultures, and radiological examinations (chest X-ray). As reported in previous studies [6, 7], the systemic immune-inflammation index (SII) was calculated using the following formula: SII = platelets × neutrophils/lymphocytes (×10^9/L). The albumin-to-fibrinogen ratio (AFR) was derived by dividing fibrinogen by albumin, whereas the neutrophil-to-lymphocyte ratio (NLR) was derived by dividing the number of neutrophils by lymphocytes.

### Statistical analysis

Statistical analyses were conducted using SPSS (version 23.0) and GraphPad (version 9.0) software, employing t-tests, Mann-Whitney U tests, and chi-square tests. Online receiver operator characteristic (ROC) curves [8] were used to evaluate the predictive value of the quantitative data for postoperative infectious complications. Binary logistic regression analysis was employed to explore independent risk factors, which were then incorporated into a nomogram predictive model using R (version 4.0.1). A two-sided *P*<0.05 was considered statistically significant.

## Results

Based on the inclusion and exclusion criteria, 301 patients who had undergone laparoscopic hysterectomy for cervical cancer were enrolled. Within one month postoperatively, 38 patients experienced infectious complications, resulting in an overall incidence rate of 12.6%. Table 1 presents the demographic, clinical, and laboratory variables associated with postoperative infectious complications. No statistically significant differences were observed in the presence of pausimenia, pathological type, FIGO stage, history of abdominal surgery, comorbidities of hypertension and COPD, or lymph node dissection between patients with and without infectious complications (*P* > 0.05). Patients ≥ 60 years of age (*P* = 0.019), BMI ≥ 24.0 (*P* = 0.001), or with diabetes (*P* = 0.007) showed a significant association with the development of postoperative infectious complications. Additionally, operation time ≥ 3 h (*P* < 0.001) and CRP level ≥ 8 mg/L (*P* = 0.035) correlated with an increased risk of infectious complications. Furthermore, patients in the complication group exhibited significantly higher levels of SII (*P* < 0.001) and NLR (*P* < 0.001) and lower levels of AFR (*P* < 0.001) thanthose in the non-complication group.

**Table 1:** Demographic and clinical variables associated with postoperative infectious complications

	Infectious complications		
Variables	Yes (*n* = 38)	No (*n* = 263)	*P*-value
Age (year)	-	-	0.019*
≥ 60	11 (28.9)	37 (14.1)	-
< 60	27 (71.1)	226 (85.9)	-
BMI (kg/m^2^)	-	-	0.001*
≥ 24.0	16 (42.1)	48 (18.3)	-
< 24.0	22 (57.9)	215 (81.7)	-
Pausimenia, n (%)	15 (39.5)	80 (30.4)	0.262
Pathological type, n (%)	-	-	0.358
Squamous cell carcinoma	34 (89.5)	246 (93.5)	-
Adenocarcinoma	4 (10.5)	17 (6.5)	-
FIGO stage, n (%)	-	-	0.498
I	29 (76.3)	213 (81.0)	-
II	9 (23.7)	50 (19.0)	-
ASA score, n (%)	-	-	0.035*
I/II	27 (71.1)	223 (84.5)	-
III	11 (28.9)	40 (15.2)	-
Comorbidities, n (%)	-	-	-
Hypertension	6 (15.8)	43 (16.3)	0.930
Diabetes	13 (34.2)	42 (16.0)	0.007*
COPD	4 (10.5)	20 (7.6)	0.534
History of abdominal surgery	6 (15.8)	36 (13.7)	0.727
Operation time (h), n (%)	-	-	< 0.001*
≥ 3	28 (73.7)	138 (52.5)	-
< 3	10 (26.3)	125 (47.5)	-
Lymph nodes dissection, n (%)	-	-	0.705
None	3 (7.9)	20 (7.6)	-
Part	25 (65.8)	189 (71.9)	-
All	10 (26.3)	54 (20.5)	-
Preoperative laboratory variables	-	-	-
White blood cell (×10^9^/L)	7.3 ± 1.9	7.1 ± 1.6	0.317
Hemoglobin (mg/dL)	10.7 ± 2.0	11.2 ± 1.8	0.116
CRP, n (%)	-	-	0.035*
≥ 8 mg/L	9 (23.7)	30 (11.3)	-
< 8 mg/L	29 (76.3)	233 (88.6)	-
SII	772 ± 187	602 ± 183	< 0.001*
AFR	9.0 ± 1.1	10.1 ± 1.2	< 0.001*
NLR	3.5 ± 0.5	3.0 ± 0.4	< 0.001*
MLR	0.25 ± 0.06	0.23 ± 0.06	0.057

BMI, body mass index; FIGO, International Federation of Gynecology and Obstetrics; COPD, Chronic Obstructive Pulmonary Disease; ASA, Amersican Society of Anesthesiologists; CRP, C-reactive protein; SII, systemic immune-inflammation index; AFR, albumin/fibrinogen ratio; NLR, neutrophil/lymphocyte ratio; MLR, monocyte/lymphocyte ratio. Data are expressed as mean ± SD or number with percentage. * *P* value < 0.05 by Chi-square test, t test or Mann Whitney U test

**Fig. 1:**
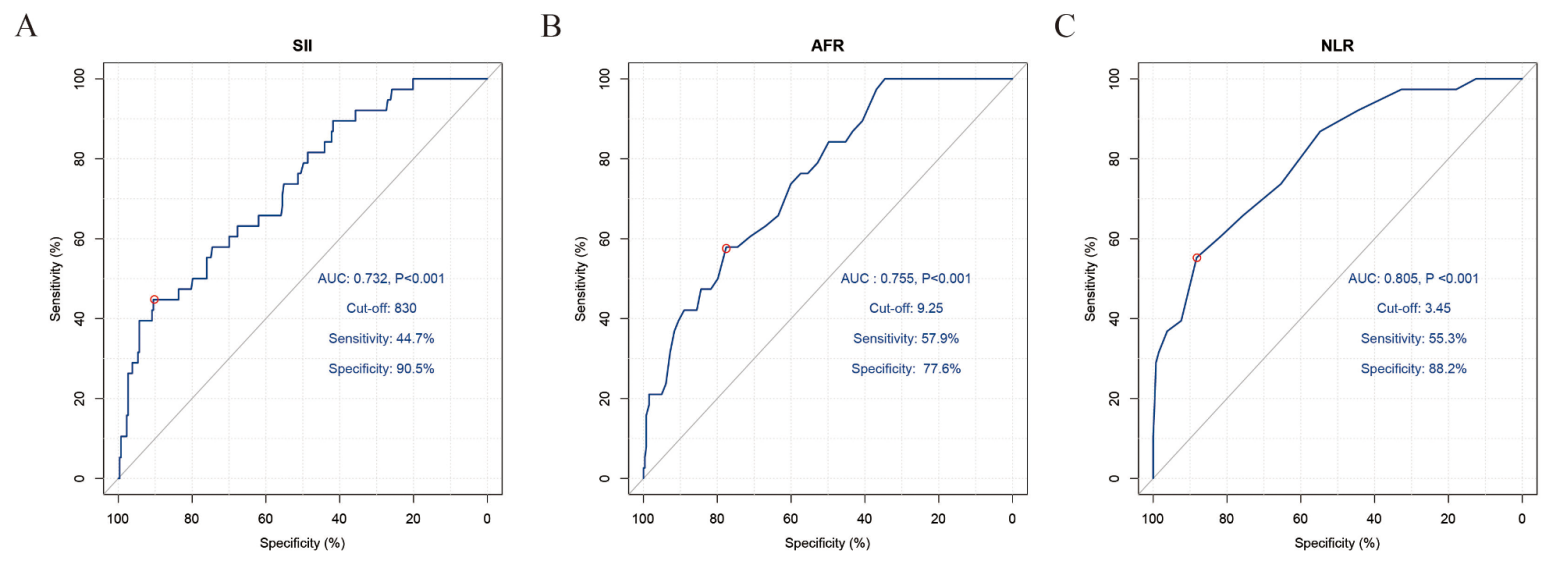
Predictive values of the SII (**A**), AFR (**B**), and NLR (**C**) for postoperative infectious complications by ROC curve analysis. ROC, receiver operating characteristic; SII, systemic immune-inflammation index; AFR, albumin/fibrinogen ratio; NLR, neutrophil-to-lymphocyte ratio; AUC, area under the curve

Three continuous variables (SII, AFR, and NLR), identified as potential risk factors for infectious complications (*P* < 0.05; Table 1), were further evaluated using ROC curves. The SII (AUC: 0.732, *P* < 0.001, cut-off value: 830), AFR (AUC: 0.755, *P* < 0.001, cut-off value: 9.25), and NLR (AUC: 0.805, *P* < 0.001, cut-off value: 3.45) were all significant predictors for postoperative infectious complications (Fig. 1). According to the cut-off points, these three continuous variables were classified into two groups: high (≥ cut-off value) and low (< cut-off value).

**Fig. 2:**
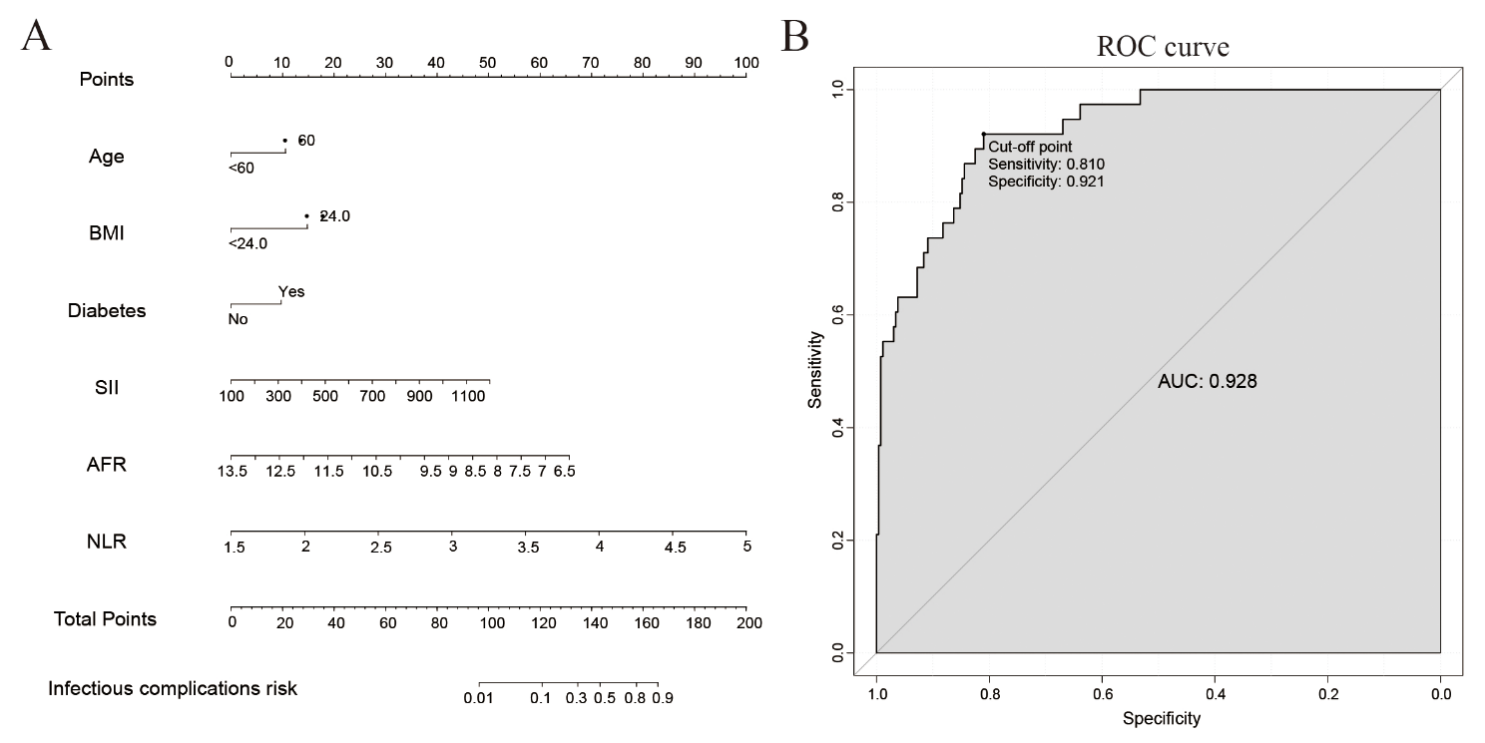
A nomogram model for postoperative infectious complications (**A**) and evaluated using ROC curve analysis (**B**). BMI, body mass index; SII, systemic immune-inflammation index; AFR, albumin-to-fibrinogen ratio; NLR, neutrophil-to-lymphocyte ratio; ROC, receiver operating characteristic; AUC, area under the curve

**Fig. 3:**
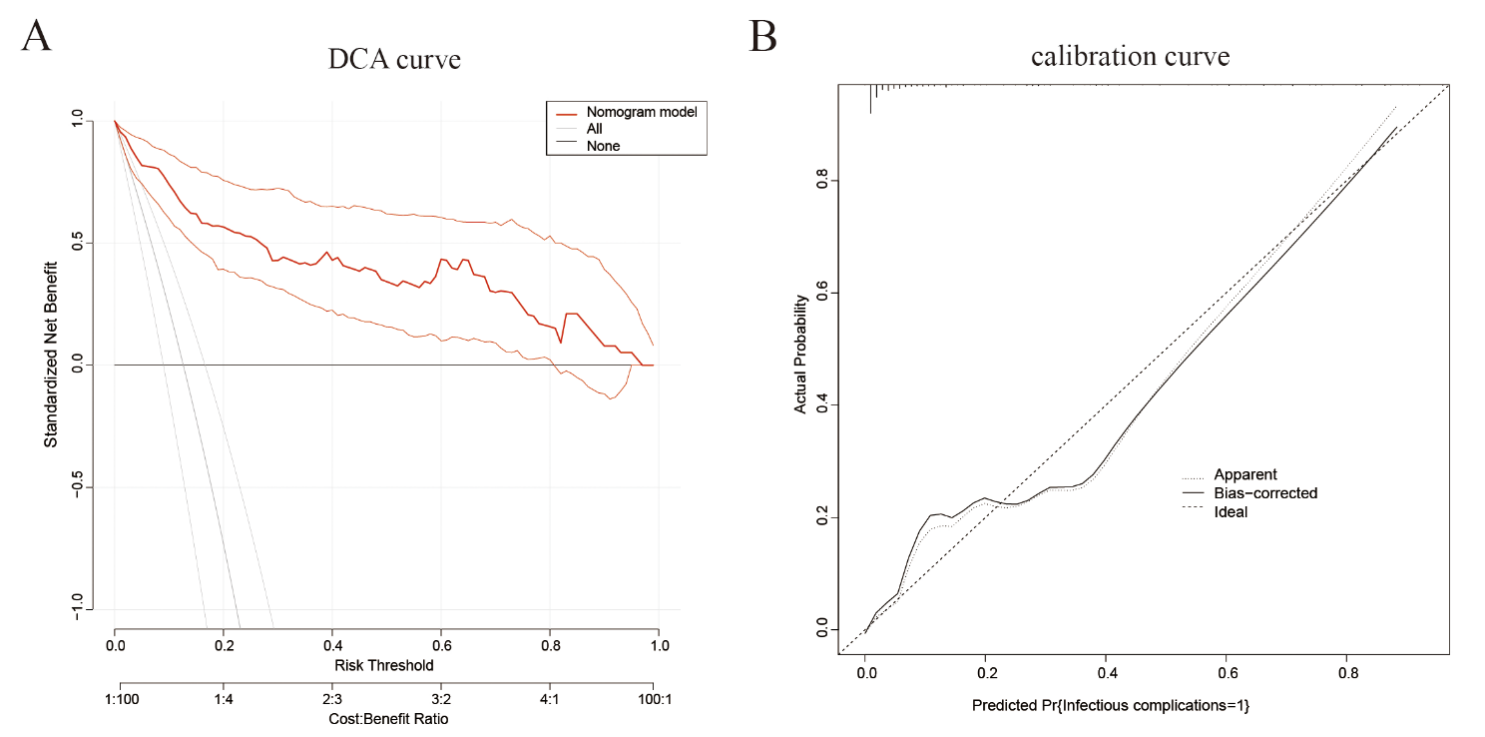
Evaluation of nomogram model using DCA (**A**) and calibration (**B**) curve analyses. DCA, decision curve analysis

Eight variables (*P* < 0.05; Table 1) were analyzed using a binary multivariate logistic regression model. Six variables were identified to be independent risk factors for postoperative infectious complications (Table 2): Age ≥ 60 (OR: 3.06, 95%CI: 1.06–8.79, *P* = 0.038), BMI ≥ 24.0 (OR: 3.70, 95%CI: 1.48–9.26, *P* = 0.005), diabetes (OR: 2.91, 95% CI: 1.10–7.73, *P* = 0.032), SII ≥ 830 (OR: 6.95, 95%CI: 2.53–19.07, *P* < 0.001), AFR < 9.25 (OR: 4.94, 95%CI: 2.02–12.07, *P* < 0.001), and NLR ≥ 3.45 (OR: 7.53, 95%CI: 3.04–18.62, *P* < 0.001). Furthermore, these six factors were incorporated into a nomogram prediction model using R The scores of SII, AFR, and NLR were significantly higher than those of age, BMI, and diabetes, indicating that they carry more weight in the predictive model (Fig. 2A). Evaluation of the nomogram model by ROC curve analysis indicated an AUC of 0.928, a sensitivity of 81.0%, and a specificity of 92.1%, respectively (Fig. 2B). Moreover, the decision curve analysis (DCA) demonstrated that this model provides superior benefits for predicting infectious complications in comparison with the “treat all” or “treat none” strategies (Fig. 3B). Furthermore, the calibration curve confirmed that the nomogram predictions (actual and bias-corrected curves) were closely aligned with the ideal curve (Fig. 3C). These findings support the strong predictive performance of our nomogram model.

**Table 2:** Binary multivariate logistic regression analysis of postoperative infectious complications

Variables	Multivariate	
	OR (95%CI)	*P* value
Age (≥ 60 vs. < 60)	3.06(1.06–8.79)	0.038*
BMI (≥ 24.0 vs. < 24.0)	3.70(1.48–9.26)	0.005*
ASA score (III vs. I/II)	1.17(0.37–3.63)	0.792
Diabetes (yes vs. no)	2.91(1.10–7.73)	0.032*
Operation time (≥ 3 vs. < 3)	1.65(0.66–4.12)	0.283
SII (≥ 830 vs. < 830)	6.95(2.53–19.07)	< 0.001*
AFR (< 9.25 vs. ≥ 9.25)	4.94(2.02–12.07)	< 0.001*
NLR (≥ 3.45 vs. < 3.45)	7.53(3.04–18.62)	< 0.001*

BMI, body mass index; ASA, Amersican Society of Anesthesiologists; SII, systemic immune-inflammation index; AFR, albumin/fibrinogen ratio; NLR, neutrophil/lymphocyte ratio; OR, odds ratio; CI, confidence interval. * *P* value < 0.05

## Discussion

Cervical cancer has become a global health threat, and its early diagnosis and treatment are crucial for patient prognosis. Several studies support the use of specific biomarkers (carcinoembryonic antigen, squamous cell carcinoma antigen, and CD44) to identify early-stage cervical cancer and, therefore, offer a better prognosis for patients [9, 10]. Infectious complications are very common postoperative complications after cervical cancer, which severely affect patients’ outcomes [11]. Consequently, identifying predictive factors for infectious complications is extremely important. The incidence of infectious complications among patients enrolled in this study was 12.6%, which is higher than the 5.8% reported by Capozzi’s group [12]. A previous study reported variable incidence rates of infectious complications after hysterectomy: 13.0% in vaginal hysterectomy, 10.5% in abdominal hysterectomy, and 9.0% in laparoscopic hysterectomy [13]. As the reported incidence of complications varies with different surgical approaches, this study chose to focused on laparoscopic hysterectomy for cervical cancer.

Despite these advancements, hysterectomy remains a compelling and challenging area of research for the surgical teams. Minimally invasive approaches have the following advantages: reduced trauma associated with surgery in terms of hospital stay, recovery time, and aesthetic outcomes. Given the improvements in patient outcomes and benefits to healthcare systems, minimally invasive surgery should be preferred when feasible and safe [14]. Some studies have shown that different surgical approaches can lead to varying complications and outcomes in the treatment of cervical cancer. A recent meta-analysis found that laparoscopic radical hysterectomy is associated with a higher risk of perioperative urologic complications compared to abdominal radical hysterectomy [15]. However, Pecorino et al. reported no significant differences in intraoperative and postoperative complication rates, follow-up death, or recurrence rates between patients who underwent abdominal or laparoscopic radical hysterectomy [16]. Another study by Corrado et al. also revealed no statistically significant difference regarding disease-free survival and overall survival between minimally invasive and abdominal radical hysterectomy [17]. Considering the potential impact of different surgical approaches on the results, we only included, patients who underwent laparoscopic hysterectomy for cervical cancer.

This study identified six independent risk factors (age ≥ 60, BMI ≥ 24.0, diabetes, SII ≥ 830, AFR < 9.25, and NLR ≥ 3.45) for postoperative infectious complications. Age is a well-recognized risk factor for postoperative complications (including infectious complications), particularly in patients aged > 60 years old [18]. Elderly patients, often experience a decline in immune function, impairing their body’s ability to fight infections. Additionally, elderly patients are more likely to have comorbid conditions (e.g., diabetes, cardiovascular diseases, and reduced renal function), which can further compromise their immune responses and wound-healing capabilities [19]. BMI ≥ 24.0 (overweight or obese in China) is usually associated with many physiological changes that can compromise surgical recovery and increase the likelihood of infections [20]. A previous study by Mullen et al. [21] showed that patients with a BMI of 25 or higher had a significantly higher rate of surgical site infections following various surgical procedures, consistent with our results. Diabetes has also been widely recognized as a risk factor for postoperative infectious complications due to its effects on the immune system and wound healing processes [22, 23]. Diabetes is associated with both microvascular and macrovascular alterations that impair blood flow, leading to decreased oxygen and nutrient supply to tissues, which are critical for healing [24, 25]. Additionally, hyperglycemia can inhibit various immune functions, including neutrophil activity, which is crucial for preventing and controlling infections [26].

The SII has emerged as a potential biomarker for predicting inflammatory status and has been associated with outcomes in various medical conditions, including cancer [27] and cardiovascular diseases [28]. A higher SII level usually indicates a pro-inflammatory state, and an imbalanced immune response, potentially leading to increased susceptibility to infections or a weakened immune defense mechanism [29, 30]. An emerging biomarker, AFR, is calculated using albumin and fibrinogen levels, and it evaluates the balance between nutritional status and the inflammatory response. AFR has been investigated in various clinical settings to predict outcomes, including cancer prognosis [31] and cardiovascular events [32]. A study by Maimaiti et al. [33] indicated that preoperative AFR can effectively predict septic failure and periprosthetic joint infection in patients who underwent revision total joint arthroplasty. All these studies support the predictive value of a low AFR level for postoperative infectious complications in this study. A widely researched biomarker, NLR, measures the balance between neutrophils and lymphocytes in the blood and has become an important prognostic indicator in various clinical diseases, including sepsis [34], *Mycoplasma* pneumoniae pneumonia [35], and cancer [36]. Neutrophils are key players in acute inflammatory responses and infections, whereas lymphocytes represent the regulatory elements of the immune system [37]. Accordingly, an elevated NLR often suggests a heightened inflammatory state and a potentially compromised immune response [37], which can predispose patients to infections.

Based on these six independent risk factors, we constructed a nomogram prediction model and the evaluation results indicated its accuracy in predicting postoperative infectious complications. This model can aid in identifying high-risk patients who may benefit from closer monitoring and pre-emptive interventions. Moreover, if complications arise, high-risk patients identified by the model could receive more intensive prophylactic antibiotic regimens, enhanced postoperative surveillance, and earlier interventions. Furthermore, early identification and management of potential infectious complications can lead to quicker interventions, reduced morbidity, and improved overall patient outcomes. In summary, this predictive tool can estimate a patient’s risk of developing infections after surgery, enabling personalized medical decision-making and improving patient outcomes.

The strength of this study lies in identifying six novel independent risk factors for postoperative infectious complications through multivariate analysis and constructing an effective individualized risk prediction model based on these factors. However, the limitations include the single-center retrospective nature of the study and the relatively small sample size, which may have omitted some important risk factors.

## Data Availability

No datasets were generated or analysed during the current study.
